# Inhibition of pannexin‐1 does not restore electrolyte balance in precystic *Pkd1* knockout mice

**DOI:** 10.14814/phy2.15956

**Published:** 2024-04-01

**Authors:** Wouter H. van Megen, Teun J. van Houtert, Caro Bos, Dorien J. M. Peters, Jeroen H. F. de Baaij, Joost G. J. Hoenderop

**Affiliations:** ^1^ Department of Medical Biosciences Radboud University Medical Center Nijmegen The Netherlands; ^2^ Department of Human Genetics Leiden University Medical Center Leiden The Netherlands

**Keywords:** ADPKD, calcium, kidney, magnesium, pannexin‐1

## Abstract

Mutations in *PKD1* and *PKD2* cause autosomal dominant polycystic kidney disease (ADPKD), which is characterized by the formation of fluid‐filled cysts in the kidney. In a subset of ADPKD patients, reduced blood calcium (Ca^2+^) and magnesium (Mg^2+^) concentrations are observed. As cystic fluid contains increased ATP concentrations and purinergic signaling reduces electrolyte reabsorption, we hypothesized that inhibiting ATP release could normalize blood Ca^2+^ and Mg^2+^ levels in ADPKD. Inducible kidney‐specific *Pkd1* knockout mice (iKsp‐*Pkd1* ^*−/−*^) exhibit hypocalcemia and hypomagnesemia in a precystic stage and show increased expression of the ATP‐release channel pannexin‐1. Therefore, we administered the pannexin‐1 inhibitor brilliant blue‐FCF (BB‐FCF) every other day from Day 3 to 28 post‐induction of *Pkd1* gene inactivation. On Day 29, both serum Ca^2+^ and Mg^2+^ concentrations were reduced in iKsp‐*Pkd1* ^*−/−*^ mice, while urinary Ca^2+^ and Mg^2+^ excretion was similar between the genotypes. However, serum and urinary levels of Ca^2+^ and Mg^2+^ were unaltered by BB‐FCF treatment, regardless of genotype. BB‐FCF did significantly decrease gene expression of the ion channels *Trpm6* and *Trpv5* in both control and iKsp‐*Pkd1* ^*−/−*^ mice. Finally, no renoprotective effects of BB‐FCF treatment were observed in iKsp‐*Pkd1* ^*−/−*^ mice. Thus, administration of BB‐FCF failed to normalize serum Ca^2+^ and Mg^2+^ levels.

## 1 INTRODUCTION

Autosomal dominant polycystic kidney disease (ADPKD) is the most prevalent genetic kidney disorder (Bergmann et al., 2018; Torres et al., 2007). It is characterized by the formation of fluid‐filled cysts within the kidney, which causes a decline in kidney function. Consequently, ADPKD patients often require renal replacement therapy. The majority of the ADPKD patients has mutations in *PKD1* or *PKD2*, which encode polycystin‐1 (PC1) and polycystin‐2 (PC2), respectively (Cornec‐Le Gall et al., 2018). Although the exact prevalence is unknown, case reports indicate that ADPKD patients may also present with electrolyte abnormalities, including hypomagnesemia and hypo‐ or hypercalcemia (Pietrzak‐Nowacka et al., 2013, 2015; Veeramuthumari & Isabel, 2013). However, the mechanism underlying the hypomagnesemia and hypocalcemia in APDKD remains elusive and is complicated by the fact that patients may be treated with different types of drugs, including diuretics against hypertension (Wuthrich et al., 2015).

Purinergic signaling is disturbed in ADPKD (Solini et al., 2015; Vallon et al., 2020) and increased ATP concentrations in the lumen of the cysts have been found (Wilson et al., 1999). Cystic epithelium has increased expression of purinergic receptors and ATP release channels (Arkhipov & Pavlov, 2019; Chang et al., 2011). We and others have recently shown that the mRNA and protein expression of the apical ATP release channel pannexin‐1 is upregulated in ADPKD, which is a main contributor to the increased ATP release in ADPKD (Arkhipov & Pavlov, 2019; Verschuren et al., 2020). Interestingly, inhibition of pannexin‐1 attenuated cyst progression and increased overall survival in a zebrafish model of ADPKD (Verschuren et al., 2020). These findings suggest that purinergic signaling could be a therapeutic target to reduce cyst growth in ADPKD.

Previous studies have shown that activation of P2X purinergic receptors by ATP inhibited magnesium (Mg^2+^) uptake in Madin‐Darby canine kidney cells (Dai et al., 2001). Moreover, ATP also inhibited the reabsorption of calcium (Ca^2+^) and sodium (Na^+^) in primary rabbit connecting tubules (CNTs) and cortical collecting duct tubules (Koster et al., 1996). Based on these findings, we hypothesized that increased pannexin‐1‐dependent ATP release might be involved in the renal loss of Ca^2+^ and Mg^2+^ in ADPKD, either by directly affecting electrolyte reabsorption through purinergic signaling or by stimulating early morphological alterations in the tubules.

We have previously demonstrated that inducible kidney‐specific *Pkd1* knockout mice (iKsp‐*Pkd1* ^*−/−*^) demonstrate hypomagnesemia and hypocalcemia in the precystic stage (Verschuren et al., 2018). Urinary Mg^2+^ or Ca^2+^ loss may explain the electrolyte disturbance in these mice. Therefore iKsp‐*Pkd1* ^*−/−*^ mice provide an interesting model to examine the role of purinergic signaling in renal Mg^2+^ and Ca^2+^ wasting in ADPKD. In this study, we administered the pannexin‐1 inhibitor brilliant blue‐FCF (BB‐FCF) to iKsp‐*Pkd1* ^*−/−*^ mice for 26 days (Wang et al., 2013). We subsequently measured serum and urinary electrolyte levels as well as the expression of calciotropic and magnesiotropic genes.

## 2 MATERIALS AND METHODS

### 2.1 Animal experiments

All animal experiments were approved by the local ethics committee of the Radboud University Nijmegen (RU DEC 2020‐0011‐001) and the national ethics committee of the Dutch Central Commission for Animal Experiments (AVD10300202010684).

iKsp‐*Pkd1* ^*−/−*^ mice have been characterized before (Lantinga‐van Leeuwen et al., 2007; Verschuren et al., 2018). Exclusively male mice were used here to exclude sex as a possible factor influencing renal electrolyte handling (Groenestege et al., 2006; Hsu et al., 2010; Verschuren et al., 2018).

Knockout of *Pkd1* was induced through administration of tamoxifen (150 mg/kg) (Sigma‐Aldrich, St. Louis, MO, USA, #5648) at postnatal Days 18 and 19 by oral gavage. Three days after induction of *Pkd1* knockout (i.e., postnatal Day 21) until 28 days post‐tamoxifen treatment, animals were injected every other day with an i.p. injection of BB‐FCF (50 mg/kg; Sigma‐Aldrich, St. Louis, MO, USA, #80717) based on previously published experiments with the related dye Brilliant Blue G (Bartlett et al., 2017) or vehicle (0.9% NaCl; B. Braun Melsungen AG, Melsungen, Germany). These timepoints were chosen as hypomagnesemia and hypocalcemia were present in iKsp‐*Pkd1* ^*−/−*^ at this age, while animals were still considered to be precystic (Verschuren et al., 2018).

The following four groups were included: control mice + vehicle (*n* = 5), control mice + BB‐FCF (*n* = 5), iKsp‐*Pkd1* ^*−/−*^ mice + vehicle (*n* = 7), and iKsp‐*Pkd1* ^*−/−*^ mice + BB‐FCF (*n* = 7). After birth, randomization was performed on a nest level: all animals from the same litter were randomly assigned to one of the indicated groups to prevent cross‐contamination with the tamoxifen and/or BB‐FCF. Group sizes were determined by a power calculation based on two‐way ANOVA with serum Ca^2+^ and Mg^2+^ concentrations as the primary outcome using G*Power 3.1 (Faul et al., 2009). One animal in the iKsp‐*Pkd1* ^*−/−*^ + BB‐FCF group was found dead in its cage after tamoxifen administration but prior to the BB‐FCF treatment. No signs of discomfort were noticed the previous day and no clear cause of death was visible.

Throughout the experiment, animals were housed in standard IVC cages on a 12‐h light/12‐h dark cycle in standard cages and had ad libitum access to standard rodent food (1% w/w Ca, 0.22% w/w Mg, 0.7% w/w PO_4_and 1000 IU/kg vitamin D_3_) and water. On experimental Days 21 and 28, all mice were placed in individual metabolic cages for 24 h in a temperature‐controlled (25°C) environment for urine and feces collection (Table S1). On Day 22 blood samples were obtained from all mice via cheek puncture. On Day 29, mice were anesthetized using 4% v/v isoflurane inhalation, after which blood samples were obtained through eye extraction and mice were immediately sacrificed through cervical dislocation. Once sacrificed, kidneys were extracted and weighed. Obtained tissue samples were snap‐frozen in liquid nitrogen and stored at −80°C (for protein and RNA isolation) or fixed in 4% v/v buffered paraformaldehyde (for immunohistochemistry). Urine and feces samples obtained from metabolic cages were stored at −20°C for electrolyte analysis. Serum was obtained from the collected blood samples through centrifuging for 10 min at 1000 g at 4°C after blood was left to clot for 30 min. The obtained serum samples were then transferred and stored at −20°C for electrolyte analysis.

### 2.2 Biochemical analyses

Serum and urinary Mg^2+^ concentrations were measured with a Xylidyl Blue‐I‐based colorimetric assay according to the manufacturer's protocol (Roche Diagnostics, Rotkreuz, Switzerland, #11489330216), while serum total calcium (Ca^2+^) and urinary Ca^2+^ concentrations were measured with a colorimetric assay as described before (van der Hagen et al., 2014). Prior to Ca^2+^ and Mg^2+^ measurements, fecal samples were incubated in nitric acid (>65%, Sigma‐Aldrich, Steinheim, Germany) for 10 min at room temperature and subsequently incubated for 30 min at 50°C, followed by another 3‐h incubation at room temperature. Fecal Ca^2+^ and Mg^2+^ concentrations were measured as described above. Urinary creatinine concentrations were measured using a Jaffé‐based assay according to the manufacturer's protocol (Labor+Technik, Berlin, Germany, #CR0054). Serum and urinary sodium (Na^+^), potassium (K^+^) and inorganic phosphate (P_i_), as well as blood urea nitrogen (BUN) concentrations were measured by the Department of Laboratory Medicine of the Radboudumc.

### 2.3 RNA extraction, cDNA synthesis and qPCR

RNA was extracted from kidney samples with a homogenizer (VWR, VDI 12, Darmstadt, Germany) using TRIzol (Invitrogen, Carlsbad, CA, USA, #12034977) according to the manufacturer's protocol. Following DNase treatment according to protocol (Promega, Madison, WI, USA, #M6101), cDNA was synthesized using Moloney Murine Leukemia Virus reverse transcriptase (Invitrogen, Carlsbad, CA, USA, #28025) using 1.5 μg total RNA. Then, qPCR was performed in triplicate for each sample using iQ SYBR Green (Bio‐Rad, Hercules, CA, USA, #1708887) and specific primers (Table S2). Data analysis was performed using the 2^−∆∆Ct^ method with *18s* as a reference gene.

### 2.4 Immunohistochemistry

Formalin‐fixed kidneys were embedded in paraffin and transverse sections of 5 μm were cut. Slides were deparaffinized in xylene for 10 min followed by 5 min incubation in a graded ethanol series (100% to 50%). Sections were subsequently incubated in 0.1% w/v hematoxylin monohydrate (Merck‐Millipore, Burlington, MA, USA, #115938) for 5 min, dipped in 50% v/v ethanol and then stained for 3 min in 0.225% w/v eosin Y (Merck‐Millipore, Burlington, MA, USA, #115938) and 0.025% w/v phloxin B (Merck‐Millipore, Burlington, MA, USA, #115926). Subsequent dehydration was performed using dipping in graded ethanol (50% to 100% v/v) and xylene, afterwards mounting was performed using Pertex (Histolabs Products AB, Västra Frölunda, Sweden, #00801).

### 2.5 Statistical analyses

Statistical analyses were performed using GraphPad Prism 9 (GraphPad software, San Diego, CA, USA). Two‐way ANOVA was used for testing the effect of two variables (i.e., treatment and genotype); in case of a significant interaction term, Tukey's post hoc test was used. For all analyses, a *p* < 0.05 was considered statistically significant. Data are presented as mean ± SD.

## 3 RESULTS

### 3.1 BB‐FCF treatment did not normalize serum Ca^2+^ or Mg^2+^ concentrations in iKsp‐Pkd1^−/−^ mice

**FIGURE 1:**
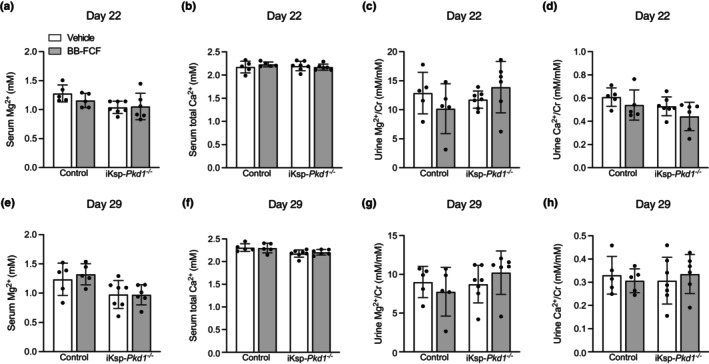
BB‐FCF treatment does not correct serum Mg^2+^ and Ca^2+^ levels in iKsp‐*Pkd1* ^*−/−*^ mice. On Day 22, serum Mg^2+^ levels were significantly reduced in iKsp‐*Pkd1* ^*−/−*^ mice. No significant effects of BB‐FCF treatment were present. *P*_genotype_ = 0.02, *P*_treatment_ = 0.4, *P*_interaction_ = 0.3 (*n* = 5–7) (a). No significant differences were present in serum total Ca^2+^ on Day 22 between any of the groups. *P*_genotype_ = 0.6, *P*_treatment_ = 0.7 *P*_interaction_ = 0.3 (*n* = 5–7) (b). Urinary Mg^2+^ excretion was not different between any of the groups on Day 22. *P*_genotype_ = 0.4, *P*_treatment_ = 0.9, *P*_interaction_ = 0.1 (*n* = 5–7) (c). No significant differences in urinary Ca^2+^ excretion were present upon BB‐FCF administration or between iKsp‐*Pkd1* ^*−/−*^ mice and control mice. *P*_genotype_ = 0.06, *P*_treatment_ = 0.09, *P*_interaction_ = 0.8 (*n* = 5–7) (d). On Day 29, serum Mg^2+^ remained significantly reduced in iKsp‐*Pkd1* ^*−/−*^ mice but was not affected by BB‐FCF treatment. *P*_genotype_ <0.01, *P*_treatment_ = 0.7, *P*_interaction_ = 0.6 (*n* = 5–7) (e). Serum total Ca^2+^ was significantly reduced in iKsp‐*Pkd1* ^*−/−*^ mice on Day 29, but not affected by BB‐FCF administration. *P*_genotype_ <0.01, *P*_treatment_ = 0.8, *P*_interaction_ = 0.6 (*n* = 5–7) (f). Urinary Mg^2+^ excretion was not significantly different between genotypes and/or treatment groups. *P*_genotype_ = 0.3, *P*_treatment_ = 0.9, P_interaction_ = 0.2 (*n* = 5–7) (g). No significant differences in urinary Ca^2+^ excretion were present between any of the groups. *P*_genotype_ = 0.9, *P*_treatment_ = 0.9, P_interaction_ = 0.5 (*n* = 5–7) (h). Data are shown as mean ± SD and analyzed by two‐way ANOVA.

To test if increased pannexin‐1 activity underlies the hypocalcemia and hypomagnesemia observed in iKsp‐*Pkd1* ^*−/−*^ mice, mice were treated with the pannexin‐1 inhibitor BB‐FCF for 26 days. Significantly lower serum Mg^2+^ concentrations (approximately 15%) were found in iKsp‐*Pkd1* ^*−/−*^ mice on day 22 (*P*_genotype_ = 0.02), but these were not altered by BB‐FCF treatment (Figure 1a). In contrast, the serum total Ca^2+^ concentration on Day 22 was not statistically different between any of the groups (Figure 1b). Urinary Mg^2+^ excretion (Figure 1c) or urinary Ca^2+^ excretion (Figure 1d) on Day 22 was also similar in both genotypes and treatment groups. One week later, serum Mg^2+^ was still significantly lower (approximately 20%) in iKsp‐*Pkd1* ^*−/−*^ mice (*P*_genotype_ = 0.004), but similar in vehicle‐ and BB‐FCF‐treated animals (Figure 1e). In addition, serum total Ca^2+^ levels were significantly lower by approximately 5% in iKsp‐*Pkd1* ^*−/−*^ mice (*P*_genotype_ = 0.004) on Day 29, in the absence of a significant effect of treatment (Figure 1f). Urinary Mg^2+^ excretion (Figure 1g) or urinary Ca^2+^ excretion (Figure 1h) were also similar in vehicle‐ and BB‐FCF treated animals on Day 29. Additionally, urinary Mg^2+^ or Ca^2+^ excretion was comparable in control and iKsp‐*Pkd1* ^*−/−*^ mice. On Day 22, fecal Mg^2+^ (Figure S1A) or Ca^2+^ (Figure S1B) excretion was unaltered in iKsp‐*Pkd1* ^*−/−*^ mice and after BB‐FCF treatment. The same trends were observed for fecal Mg^2+^ excretion (Figure S1C) and fecal Ca^2+^ excretion (Figure S1D) on Day 29.

Serum Na^+^ was not altered by treatment or genotype, while urinary Na^+^ excretion was significantly reduced in iKsp‐*Pkd1* ^*−/−*^ mice (*P*_genotype_ = 0.04) (Figure S2A,B). The serum K^+^ concentration was significantly increased upon BB‐FCF treatment (approximately 2% in control mice and 13% in iKsp‐*Pkd1* ^*−/−*^ mice) in the absence of significant differences in urinary K^+^ excretion between any of the groups (Figure S2C,D).

### 3.2 BB‐FCF treatment did not alter gene expression involved in paracellular Ca^2+^ or Mg^2+^ reabsorption

**FIGURE 2:**
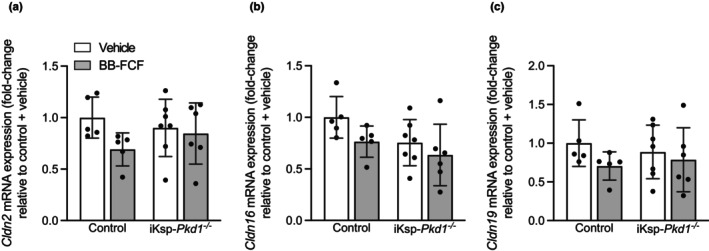
Unaltered expression of claudins involved in paracellular Ca^2+^ and Mg^2+^ reabsorption. On Day 29, no significant differences were present in *Cldn2* between any of the groups. *P*_genotype_ = 0.8, *P*_treatment_ = 0.1, *P*_interaction_ = 0.2 (*n* = 5–7) (a). *Cldn16* expression was also not different between genotypes and/or treatment groups. *P*_genotype_ = 0.07, *P*_treatment_ = 0.08, *P*_interaction_ = 0.6 (*n* = 5–7) (b). The expression of *Cldn19* was not significantly different between any of the groups. *P*_genotype_ = 0.9, *P*_treatment_ = 0.2, *P*_interaction_ = 0.5 (*n* = 5–7) (c). Data are shown as mean ± SD and analyzed by two‐way ANOVA.

Next, we determined if pannexin‐1 inhibition influenced the expression of genes involved in renal Ca^2+^ and Mg^2+^ reabsorption. The expression of *Cldn2*, involved in paracellular Ca^2+^ reabsorption (Muto et al., 2010), was not significantly different between treatment groups or genotypes (Figure 2a). The expression *Cldn16* (Figure 2b) and *Cldn19* (Figure 2c), which facilitate paracellular Ca^2+^ and Mg^2+^ reabsorption in the thick ascending limb of Henle's loop (TAL), was also similar between the four different groups (Konrad et al., 2006; Olinger et al., 2018; Simon et al., 1999). This suggests that pannexin‐1 inhibition did not alter Ca^2+^ and Mg^2+^ reabsorption in these segments.

### 3.3 BB‐FCF treatment reduced expression of genes involved in transcellular Mg^2+^ reabsorption

**FIGURE 3:**
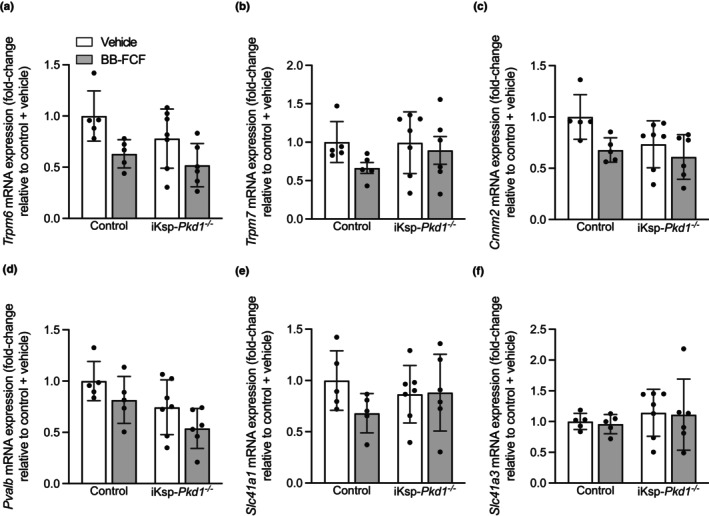
Effects of BB‐FCF treatment on renal magnesiotropic gene expression in iKsp‐Pkd1^*−/−*^ mice. On Day 29, *Trpm6* expression was significantly decreased in both control and iKsp‐*Pkd1* ^*−/−*^ mice after BB‐FCF treatment. *P*_genotype_ = 0.1, *P*_treatment_ <0.01, *P*_interaction_ = 0.6 (*n* = 5–7) (a). *Trpm7* expression was not significantly different between any of the groups. *P*_genotype_ = 0.5, *P*_treatment_ = 0.2, *P*_interaction_ = 0.4 (*n* = 5–7) (b). *Cnnm2* expression was significantly decreased by BB‐FCF treatment but not by genotype. *P*_genotype_ = 0.07, *P*_treatment_ = 0.02, *P*_interaction_ = 0.3 (*n* = 5–7) (c). The expression of *Pvalb* was significantly lower in iKsp‐*Pkd1* ^*−/−*^ mice compared to control animals, while no significant effect of treatment was present. *P*_genotype_ = 0.01, *P*_treatment_ = 0.05, *P*_interaction_ = 0.9 (*n* = 5–7) (d). *Slc41a1* expression was not significantly changed by genotype or treatment. *P*_genotype_ = 0.8, *P*_treatment_ = 0.2, *P*_interaction_ = 0.2 (*n* = 5–7) (e). The expression of *Slc41a3* was also not significantly altered by genotype or treatment. *P*_genotype_ = 0.4, *P*_treatment_ = 0.8, *P*_interaction_ = 0.9 (*n* = 5–7) (f). Data are shown as mean ± SD and analyzed by two‐way ANOVA.

In the distal convoluted tubule (DCT), Mg^2+^ is reabsorbed transcellularly through apical uptake by transient receptor potential 6 (TRPM6) and transient receptor potential melastatin 7 (TRPM7) (de Baaij, 2023; Ferioli et al., 2017; Voets et al., 2004), while also parvalbumin, cyclin M2 (CNNM2), solute carrier family 41 type A1 (SLC41A1) and solute carrier family 41 type A3 (SLC41A3) have been implicated in Mg^2+^ reabsorption in this segment (de Baaij, 2023; de Baaij, Arjona, et al., 2016; Hirata et al., 2014; Kolisek et al., 2008; Olinger et al., 2012; Stuiver et al., 2011). A significant decrease in renal *Trpm6* expression (approximately 35%) was observed upon BB‐FCF treatment in both genotypes (*P*_treatment_ = 0.005) (Figure 3a), while *Trpm7* expression was unchanged by treatment or genotype (Figure 3b). The expression of *Cnnm2* was significantly decreased by BB‐FCF treatment (*P*_treatment_ = 0.02) (Figure 3c). *Pvalb* expression was significantly lower in iKsp‐*Pkd1* ^*−/−*^ mice compared to control mice, but no significant differences were present between vehicle‐ and BB‐FCF‐treated animals (*P*_genotype_ = 0.01) (Figure 3d). *Slc41a1* (Figure 3e) and *Slc41a3* (Figure 3f) expression was comparable between all groups. Thus, BB‐FCF reduced the expression of several genes involved in Mg^2+^ reabsorption in the DCT independent of genotype.

### 3.4 BB‐FCF treatment decreased gene expression of proteins involved in transcellular Ca^2+^ reabsorption

**FIGURE 4:**
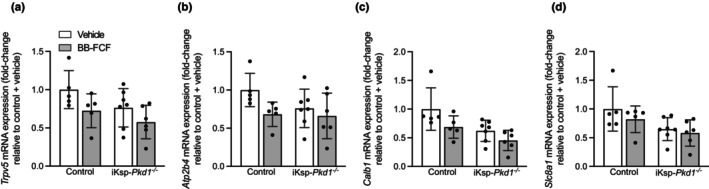
Effects of BB‐FCF treatment on renal calciotropic gene expression in iKsp‐Pkd1^*−/−*^ mice. On Day 29, T*rpv5* expression was significantly decreased after BB‐FCF treatment compared to vehicle‐treated animals, but not affected by genotype. *P*_genotype_ = 0.07, *P*_treatment_ = 0.03, *P*_interaction_ = 0.7 (*n* = 5–7) (a). *Atp2b4* expression was not significantly altered by treatment or genotype. *P*_genotype_ = 0.2, *P*_treatment_ = 0.06, *P*_interaction_ = 0.3 (*n* = 5–7) (b). *Calb1* expression was significantly decreased in iKsp‐*Pkd1* ^*−/−*^ mice compared to control animals and after BB‐FCF treatment. *P*_genotype_ <0.01, *P*_treatment_ = 0.03, *P*_interaction_ = 0.5 (*n* = 5–7) (c). *Slc8a1* expression was significantly lower in iKsp‐*Pkd1* ^*−/−*^ mice compared to control animals. *P*_genotype_ = 0.02, *P*_treatment_ = 0.3, *P*_interaction_ = 0.6 (*n* = 5–7) (d). Data are shown as mean ± SD and analyzed by two‐way ANOVA.

Transcellular Ca^2+^ reabsorption in the DCT and CNT is dependent on apical uptake by transient receptor potential vanilloid 5 (TRPV5), shuttling by calbindin‐D_28k_ (*Calb1*) and basolateral extrusion by Na^+/^Ca^2+^ exchanger 1 (NCX1; *Slc8a1*) and plasma membrane Ca^2+^‐ATPase 4 (PMCA4; *Atp2b4*) (Hoenderop et al., 1999, 2000; Kojetin et al., 2006; Magyar et al., 2002; Moor & Bonny, 2016; van der Hagen et al., 2014). *Trpv5* expression was approximately 25% lower after BB‐FCF treatment in both genotypes, while no significant differences were present between control and iKsp‐*Pkd1* ^*−/−*^ mice (*P*_treatment_ = 0.03) (Figure 4a). *Atp2b4* expression was similar in all treatment groups and genotypes (Figure 4b). Furthermore, *Calb1* expression was significantly lower in iKsp‐*Pkd1* ^*−/−*^ mice and also reduced by BB‐FCF treatment (*P*_treatment_ = 0.03, *P*_genotype_ = 0.006) (Figure 4c). Finally, iKsp‐*Pkd1* ^*−/−*^ mice exhibited significantly lower *Slc8a1* expression, but the effect of treatment was not significantly different (*P*_genotype_ = 0.02) (Figure 4d). These results show that BB‐FCF reduced the gene expression of several calciotropic genes in the DCT/CNT in both control and iKsp‐*Pkd1* ^*−/−*^ mice.

### 3.5 Effect of BB‐FCF treatment on renal P_i_ handling

**FIGURE 5:**
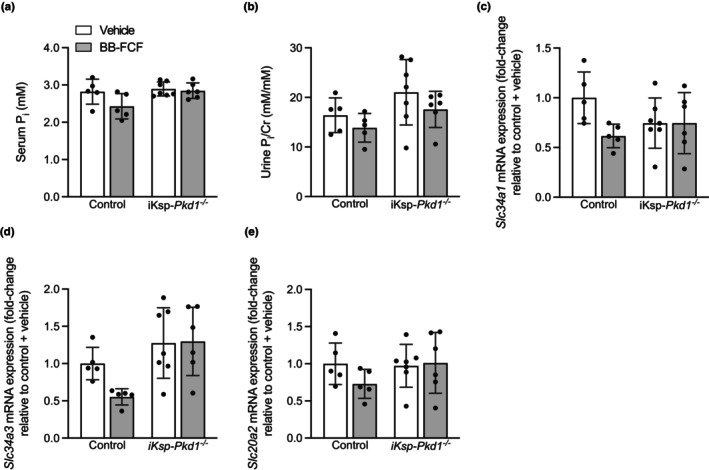
Effects of BB‐FCF treatment on renal phosphate handling in iKsp‐Pkd1^*−/−*^ mice. On Day 29, the serum P_i_ concentration was significantly higher in iKsp‐*Pkd1* ^*−/−*^ mice compared to control animals. *P*_genotype_ = 0.046, *P*_treatment_ = 0.07, *P*_interaction_ = 0.2 (*n* = 5–7) (a). Urinary P_i_ excretion on Day 29 was significantly higher in iKsp‐*Pkd1* ^*−/−*^ mice compared to control animals. *P*_genotype_ = 0.046, *P*_treatment_ = 0.1, *P*_interaction_ = 0.8 (*n* = 5–7) (b). *Slc34a1* expression was not significantly altered by treatment or genotype. *P*_genotype_ = 0.6, *P*_treatment_ = 0.08, *P*_interaction_ = 0.08 (*n* = 5–7) (c). *Slc34a3* expression was significantly increased in iKsp‐*Pkd1* ^*−/−*^ mice compared to control animal, but not significantly affected by BB‐FCF treatment. *P*_genotype_ <0.01, *P*_treatment_ = 0.2, *P*_interaction_ = 0.2 (*n* = 5–7) (d). *Slc20a2* expression was not significantly different between any of the groups. *P*_genotype_ = 0.3, *P*_treatment_ = 0.4, *P*_interaction_ = 0.3 (*n* = 5–7) (e). Data are shown as mean ± SD and analyzed by two‐way ANOVA.

Serum P_i_ was significantly increased in iKsp‐*Pkd1* ^*−/−*^ mice (*P*_genotype_ = 0.04) (Figure 5a). This was paralleled by a significant increase in urinary P_i_ excretion in iKsp‐*Pkd1* ^*−/−*^ mice compared to control mice (*P*_genotype_ = 0.046) (Figure 5b). Renal P_i_ reabsorption occurs for 80% in the proximal tubule and is mediated by the apical transporters Na^+^‐dependent phosphate transporter 2A (Napi2a; encoded by *Slc34a1*), Na^+^‐dependent phosphate transporter 2C (Napi2c; encoded by *Slc34a3*) and Na^+^‐dependent phosphate transporter 2 (Pit‐2; encoded by *Slc20a2*) (Alexander & Dimke, 2023). *Slc34a1* expression was not significantly different between control and iKsp‐*Pkd1* ^*−/−*^ mice or between vehicle‐ and BB‐FCF‐treated animals (Figure 5c). *Slc34a2* expression was significantly higher in iKsp‐*Pkd1* ^*−/−*^ mice compared to control animals (*P*_genotype_ = 0.004) (Figure 5d). *Slc20a2* expression was not affected by genotype nor by treatment (Figure 5e). Together, these results indicate that the increase urinary P_i_ excretion is not explained by alterations in gene expression of renal P_i_ transporters and may instead reflect the increased serum P_i_ concentrations.

### 3.6 BB‐FCF treatment did not alter precystic parameters in iKsp‐Pkd1 ^−/−^ mice

**FIGURE 6:**
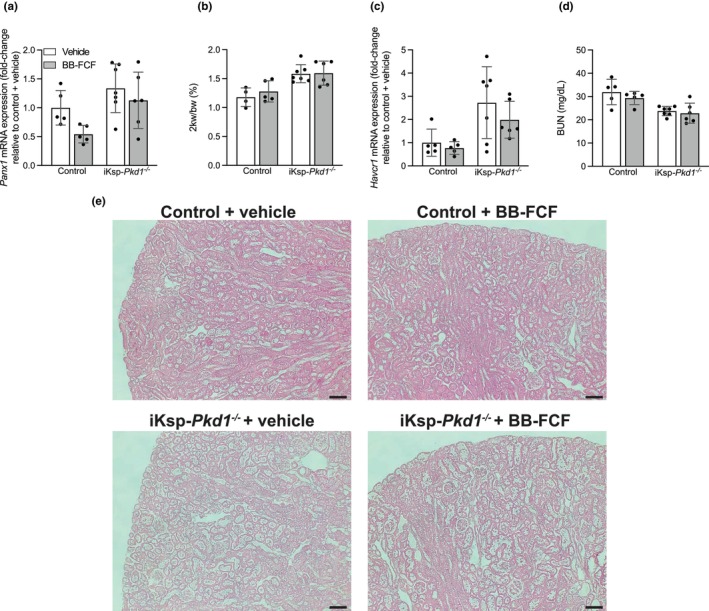
Effects of BB‐FCF treatment on precystic parameters of iKsp‐*Pkd1* ^*−/−*^ mice. Renal *Panx1* expression was significantly higher in iKsp‐*Pkd1* ^*−/−*^ mice and significantly decreased after BB‐FCF treatment. *P*_genotype_ <0.01, *P*_treatment_ = 0.049, *P*_interaction_ = 0.4 (*n* = 5–7) (a). The weight of the two kidneys relative to body weight was significantly higher in iKsp‐*Pkd1* ^*−/−*^ mice. *P*_genotype_ <0.001, *P*_treatment_ = 0.5, *P*_interaction_ = 0.6 (*n* = 4–7) (b). *Havcr1* expression was significantly increased in iKsp‐*Pkd1* ^*−/−*^ mice but not affected by treatment. *P*_genotype_ <0.01, *P*_treatment_ = 0.3, *P*_interaction_ = 0.6 (*n* = 5–7) (c). The concentration of blood urea nitrogen (BUN) was significantly lower in iKsp‐*Pkd1* ^*−/−*^ mice compared to control animals. *P*_genotype_ <0.001, *P*_treatment_ = 0.3, *P*_interaction_ = 0.6 (*n* = 5–7) (d). Hematoxylin and eosin staining of kidneys showed mild tubular dilation in iKsp‐*Pkd1* ^*−/−*^ mice, which was not altered upon BB‐FCF treatment. Scalebar indicates 100 μm (e). Data are shown as mean ± SD and analyzed by two‐way ANOVA.

*Panx1* expression was significantly increased in iKsp‐*Pkd1* ^*−/−*^ mice (*P*_genotype_ = 0.009) (Figure 6a). Interestingly, a significant decrease in *Panx1* expression after BB‐FCF treatment was present in both control mice and iKsp‐*Pkd1* ^*−/−*^ mice (*P*_treatment_ = 0.049). A significant increase in two kidneys weight to body weight was present in iKsp‐*Pkd1* ^*−/−*^ mice, which was not significantly altered by BB‐FCF treatment (*P*_genotype_ = 0.0002) (Figure 6b). The expression of *Havcr1*, which encodes kidney‐injury molecule‐1 (Kim‐1), a marker of kidney tubular damage (Ichimura et al., 2004), was also significantly increased in iKsp‐*Pkd1* ^*−/−*^ mice compared to control animals (*P*_genotype_ = 0.003), while no significant effect of treatment was present (Figure 6c). However, despite these signs of kidney damage, BUN was not increased in iKsp‐*Pkd1* ^*−/−*^ mice (Figure 6d). In fact, BUN levels were significantly lower in iKsp‐*Pkd1* ^*−/−*^ mice (*P*_genotype_ = 0.0002). Representative hematoxylin and eosin staining of kidney tissue showed dilated tubules in the cortex of iKsp‐*Pkd1* ^*−/−*^ mice, which appeared unaltered after BB‐FCF treatment (Figure 6e).

## 4 DISCUSSION

In the current study, we show that pannexin‐1‐dependent ATP release is unlikely to explain the hypomagnesemia and hypocalcemia in iKsp‐*Pkd1* ^*−/−*^ mice. This conclusion is based on the following observations: (i) administration of BB‐FCF did not normalize the low serum Mg^2+^ and Ca^2+^ concentrations in iKsp‐*Pkd1* ^*−/−*^ mice; (ii) genes involved in Mg^2+^ and Ca^2+^ reabsorption in the kidney were downregulated in iKsp‐*Pkd1* ^*−/−*^ mice independent of BB‐FCF treatment; (iii) kidney histology was similar in BB‐FCF‐ and vehicle‐treated animals, indicating that increased pannexin‐1 activity might not indirectly reduce renal electrolyte transport through altering kidney morphology.

Purinergic signaling is an important regulator of renal electrolyte reabsorption (Arkhipov & Pavlov, 2019; Dai et al., 2001; Gailly et al., 2014; Koster et al., 1996; Stockand et al., 2010; Vallon et al., 2020). Previous studies reported that extracellular ATP inhibits Mg^2+^ and Ca^2+^ transport in vitro (Dai et al., 2001; Koster et al., 1996). In contrast, we demonstrate that ATP release via pannexin‐1 is likely not involved in the previously reported effects of extracellular ATP on mineral reabsorption. Although our study does not exclude pannexin‐1 independent effects, it should be noted that in vivo evidence for the effects of extracellular ATP on Mg^2+^ and Ca^2+^ transport is lacking to date. Indeed, all previous studies in mice did not support an effect of purinergic signaling on electrolyte reabsorption. For instance, serum and urinary levels of Mg^2+^ and Ca^2+^ were not significantly different in P2X6 knockout mice (de Baaij, Kompatscher, et al., 2016). Additionally, P2X4 knockout mice did not exhibit alterations in urinary or serum Mg^2+^ levels (de Baaij et al., 2014). These findings are in line with the unaltered renal excretion of Mg^2+^ and Ca^2+^ in both control and iKsp‐*Pkd1* ^*−/−*^ mice after BB‐FCF treatment.

Our results consistently demonstrate that BB‐FCF did not modify Mg^2+^ and Ca^2+^ levels in blood and urine at Day 22 and 29, implying that pannexin‐1 is not involved in the regulation of mineral reabsorption in the kidney. Nevertheless, it is important to consider the validity of the model, the efficiency of the treatment and the potential compensating mechanisms that would mask the effects of BB‐FCF.

First, iKsp‐*Pkd1* ^*−/−*^ mice are an established model to study electrolyte levels in the context of ADPKD (Verschuren et al., 2018). The mice display low serum Mg^2+^ and Ca^2+^ levels in the precystic phase. Although we did not observe frank hypercalciuria or hypermagnesiuria on any of the time points in iKsp‐*Pkd1* ^*−/−*^ mice, the similar urinary Mg^2+^ and Ca^2+^ excretion as control animals implies a renal leak, that is, the inability of the kidneys to increase reabsorption upon reduced serum levels. However, the observed reductions in serum Mg^2+^ and Ca^2+^ concentrations in precystic iKsp‐*Pkd1* ^*−/−*^ mice are relatively modest and may not result in symptoms associated with hypomagnesemia and hypocalcemia. It is possible that progression into a more advanced stage of ADPKD results in a more pronounced decrease in serum Mg^2+^ and Ca^2+^ levels. Future studies into the electrolyte phenotype of cystic iKsp‐*Pkd1* ^*−/−*^ mice are therefore warranted, as well as any potential effects of purinergic signaling inhibition on electrolyte homeostasis.

Second, the clear change in urinary color from yellow to green‐blueish strongly indicates that the BB‐FCF did reach the kidneys. In line with this, treatment with BB‐FCF slightly but significantly reduced *Panx1* expression in both control and iKsp‐*Pkd1* ^*−/−*^ mice. Thus, BB‐FCF clearly had biological effects in the kidney. Unfortunately, we were unable to measure urinary ATP concentrations in the current study. We collected 24 h urine in a warmed environment to reduce animal discomfort, and therefore the majority of the urinary ATP was likely already hydrolyzed by ectonucleotidases or ATPases (Gutierrez Cruz et al., 2022). As BB‐FCF is not well characterized, it is therefore unclear if the administered dose was sufficient to reduce pannexin‐1‐dependent ATP release. The administered dose was based on studies using Brilliant Blue G, which has some degree of similarity with BB‐FCF and also inhibits pannexin‐1 (Qiu & Dahl, 2009; Wang et al., 2013). In contrast to BB‐FCF, Brilliant Blue G is also known to inhibit the P2X7 receptor in the μM range (Wang et al., 2013). Furthermore, BB‐FCF has a higher affinity for pannexin‐1 than Brilliant Blue G (IC_50_: 0.27 vs. 3 μM) (Qiu & Dahl, 2009). Based on the higher potency of BB‐FCF, administration of the same dose as previously used for Brilliant Blue G is therefore likely to be sufficient for pannexin‐1 inhibition. However, measuring urinary ATP concentrations and determining the pharmacokinetics of BB‐FCF are required to determine the optimal treatment strategy for BB‐FCF‐dependent pannexin‐1 inhibition in vivo.

Nevertheless, we did observe a significant decrease in the expression of genes involved in transcellular Mg^2+^ reabsorption (*Trpm6* and *Cnnm2*) and Ca^2+^ reabsorption (*Trpv5* and *Calb1*) after BB‐FCF treatment. Despite the fact that we did not find an upregulation of genes involved in paracellular Ca^2+^ and Mg^2+^ reabsorption, nor of increased intestinal absorption given the unaltered fecal excretion, it appears that these decreases are not sufficient to compromise Ca^2+^ and Mg^2+^ balance. However, this does suggest that BB‐FCF treatment induced biological effects in the kidney. As the in vivo effects of BB‐FCF administration remain to be fully determined, we also cannot exclude that off‐target effects of BB‐FCF underlie these alterations in gene expression. As mentioned above, the specificity of BB‐FCF is not well characterized. Although BB‐FCF is described as a selective inhibitor for pannexin‐1 as it does not also inhibit the P2X7 purinergic receptor (Wang et al., 2013), it is currently unclear if BB‐FCF might have off‐target effects. For example, it remains to be determined if it can also inhibit other ATP release channels or purinergic receptors other than P2X7. Further in vitro experiments determining the effects of BB‐FCF on ATP release channel activity and purinergic receptor activity are therefore required to determine the selectivity of BB‐FCF. Characterization of basic cell parameters including cell proliferation, gene expression and metabolic activity could also aid to determine if any undesired off‐target effects of BB‐FCF exist.

Moreover, the blueish color of the feces indicated that BB‐FCF also reached the intestines. Although we cannot exclude an inhibiting effect of BB‐FCF on intestinal Ca^2+^ and Mg^2+^ absorption, it seems unlikely that a combination of reduced intestinal uptake and renal reabsorption would be insufficient to decrease serum Mg^2+^ and Ca^2+^ concentrations. Furthermore, the fact that BB‐FCF did not alter fecal Mg^2+^ and Ca^2+^ excretion indicates that it did not interfere with intestinal absorption of these electrolytes. Although our studies do not exclude changes in Mg^2+^ or Ca^2+^ storage in bone and soft tissues upon BB‐FCF treatment, it is unlikely that major shifts in the mineral balance take place when urinary and fecal excretion are unaffected.

Previous studies have shown that increased purinergic signaling is observed in ADPKD. For example, increased expression of the ATP‐release channel pannexin‐1 was present in cystic and precystic mouse models of ADPKD (Arkhipov & Pavlov, 2019; Verschuren et al., 2020). Moreover, iKsp‐*Pkd1* ^*−/−*^ mice exhibited increased urinary ATP levels compared to control mice (Verschuren et al., 2020). An increase in the expression of purinergic receptors, including P2X7, P2Y2, and P2Y6 has also been reported in rodent models mimicking ADPKD (Chang et al., 2011; Turner et al., 2004). Importantly, the pannexin‐1 inhibitor BB‐FCF reduced cyst progression and increased survival in a *pkd2* zebrafish model of ADPKD (Verschuren et al., 2020). These findings clearly illustrate that purinergic signaling is dysregulated in ADPKD.

The iKsp‐*Pkd1* ^*−/*^ mice develop renal failure around 10–12 weeks after tamoxifen administration (Leonhard, Happe, & Peters, 2016; Leonhard, Kunnen, et al., 2016). Therefore, we studied these mice in a very early stage of the disease, during which no cysts had been formed yet. Although tubular dilations of the proximal tubule and collecting duct were present (Verschuren et al., 2018), this was not the case for the TAL and DCT. Nevertheless, the previously observed decreased expression of *Umod* and *Pvalb*, markers for the TAL and early DCT, respectively, indicated tubular remodeling might underlie renal electrolyte wasting (Verschuren et al., 2018). If this is the case, this process might not be dependent on pannexin‐1‐dependent ATP release, since BB‐FCF did not affect tubule morphology in our iKsp‐*Pkd1* ^*−/−*^ mice. This is in contrast to the significantly smaller cyst size in *pkd2* knockdown zebrafish following BB‐FCF treatment compared to vehicle treatment. It is possible that differences in treatment dose underlie these differences, as the concentration that reaches the kidneys after i.p. injection in mice might differ from the concentration that reaches the zebrafish pronephros when BB‐FCF was added to the medium. Moreover, the *pkd2* zebrafish model also reflects a more rapid progression of ADPKD, as 5 days postfertilization almost 80% of the *pkd2* knockdown zebrafish in the vehicle group had already died and exhibited a large cyst in the pronephros (Verschuren et al., 2020). Since the current study used mice in an early stage of the disease, this might explain why no effects of BB‐FCF were present on kidney histology or on the expression of the injury marker *Havcr1* (Ichimura et al., 2004). We can also not exclude that additional differences between the *pkd2* zebrafish model and the iKsp‐*Pkd1* ^*−/−*^ mice contribute to the difference in response to BB‐FCF. For example, the zebrafish pronephros consists of only two nephrons with a shared glomerulus and the *pkd2* zebrafish model only forms one large cyst, in contrast to the more complex microenvironment of early cystic and non‐cystic tissue in iKsp‐*Pkd1* ^*−/−*^ mice (Drummond et al., 1998; Poureetezadi & Wingert, 2016; Verschuren et al., 2020).

Importantly, BB‐FCF treatment did also not reduce the formation of cysts in the *pkd2* zebrafish model, but instead mainly slowed cyst growth (Verschuren et al., 2020). This further suggests that BB‐FCF might not be suitable for treatment in early ADPKD.

Recent clinical trials showed promising treatment potential of the vasopressin V_2_ receptor antagonist tolvaptan in ADPKD, as indicated by the slowed decline in kidney function and occurrence of advanced stages of chronic kidney disease (Torres et al., 2012, 2017). However, tolvaptan may not be effective in patients over 55 years old or in nonwhite patients (Torres et al., 2012). Additionally, tolvaptan treatment is associated with side effects including polyuria, thirst, and liver injury (Torres et al., 2012, 2017). This warrants the investigation of additional therapeutic strategies. We previously showed a reduction in cyst progression in BB‐FCF‐treated *pkd2* knockdown zebrafish. Recently, the nonselective pannexin‐1 inhibitor probenecid reduced cyst progression in a mouse model (*Pkd1* ^*RC/RC*^) of ADPKD (Arkhipov et al., 2023). Moreover, this study also showed that probenecid slowed the decline in glomerular filtration rate. However, the effect of probenecid was restricted to male mice, as cyst progression was not significantly different in female mice. Given that female *Pkd1* ^*RC/RC*^ mice have an accelerated progression of the disease, this suggests that inhibition of pannexin‐1 might be more effective in an earlier stage of the disease (Arkhipov et al., 2023).

In contrast, the results from the current study and from our *pkd2* zebrafish model indicate that pannexin‐1 might not be effective in very early stages of the disease, in which cyst formation is still ongoing. Moreover, the increase in *Panx1* mRNA expression in our precystic iKsp‐*Pkd1* ^*−/−*^ mice is relatively modest, in contrast to the clear increase in pannexin‐1 protein expression in cells lining cystic tissue in kidney biopsies of human ADPKD patients (Arkhipov et al., 2023; Verschuren et al., 2020). Although this modest increase in *Panx1* mRNA expression in precystic ADPKD does not mean that there is no increase in pannexin‐1 activity, we speculate that the increase in pannexin‐1 expression might be more pronounced in more advanced stages of the disease. Studies into the possible treatment potential of BB‐FCF in ADPKD should therefore be performed in cystic animals. Additionally, it should be considered that the treatment duration was relatively short in our current study. For proper assessment of the therapeutic potential of BB‐FCF in ADPKD, administration of the compound for a longer period should also be considered, at least until the mice reach a more severe stage of the disease. Furthermore, to study whether BB‐FCF treatment affects existing cysts in iKsp‐*Pkd1* ^*−/−*^ mice, treatment could also be initiated at a later period in cystic mice instead of increasing treatment duration. Nevertheless, for treatment purposes probenecid or Brilliant Blue G might be more effective than BB‐FCF, as both drugs also block the P2X7 purinergic receptor, which is also involved in cyst development in ADPKD (Bhaskaracharya et al., 2014; Chang et al., 2011; Hillman et al., 2004; Qiu & Dahl, 2009; Wang et al., 2013).

In conclusion, we show here that administration of BB‐FCF did not correct the hypomagnesemia and hypocalcemia in iKsp‐*Pkd1* ^*−/−*^ mice. Future studies should therefore examine alternative therapeutic approaches to limit hypomagnesemia and hypocalcemia in ADPKD.

## AUTHOR CONTRIBUTIONS

WvM and JH conceived the research; WvM, CB, DP, JdB, and JH designed the research; WvM, TvH, and CB performed experiments; WvM and TvH analyzed the data; WvM, TvH, JdB, and JH interpreted results of experiments; WvM prepared figures; WvM, JdB, and JH drafted the manuscript; WvM, TvH, CB, DP, JdB, and JH edited, revised and approved the final version of the manuscript.

## FUNDING INFORMATION

This work was supported by a PhD research grant from the Radboud Institute for Molecular Life Sciences within the Radboud university medical center to WvM and JH.

## CONFLICT OF INTEREST STATEMENT

The authors have declared that no conflicts of interest exist.

## ETHICS STATEMENT

The animal experiments described in this manuscript were approved by the local ethics committee of the Radboud University Nijmegen (RU DEC 2020‐0011‐001) and the national ethics committee of the Dutch Central Commission for Animal Experiments (AVD10300202010684).

## Supporting information


Data S1.

## Data Availability

All data presented in the current study, as well as the raw data, are available upon reasonable request to the corresponding author.
