# Supplementary material for: Inhibition of pannexin‐1 does not restore electrolyte balance in precystic Pkd1 knockout mice
Source: Physiol Rep. 2024 Apr 1;12(7):e15956. doi: 10.14814/phy2.15956 (PMC10984814; doi:10.14814/phy2.15956)
Supplement: Supplementary file 1 — Data S1. [file PHY2-12-e15956-s001.docx]

**Supplemental information**

**Tables**

Table S1: Metabolic parameters of control and iKsp-*Pkd1^-/-^* mice.

Table S2: Primer sequences used for gene expression analysis by qPCR.

**Supplemental figures**

Figure S1: BB-FCF treatment does not change fecal Mg^2+^ and Ca^2+^ levels in iKsp-*Pkd1^-/-^* mice.

Figure S2: Serum and urinary Na^+^ and K^+^ levels after BB-FCF treatment in iKsp-*Pkd1^-/-^* mice.

**Table S1** | **Metabolic parameters of control and iKsp-Pkd1^-/-^ mice.**

|  | **Control + vehicle** | **Control +**  **BB-FCF** | **iKsp-*Pkd1^-/-^* +**  **vehicle** | **iKsp-*Pkd1^-/-^* +**  **BB-FCF** |
| --- | --- | --- | --- | --- |
| **Day 22** |  |  |  |  |
| Body weight (g) | 16.7 ± 1.9 | 18.1 ± 0.7 | 15.4 ± 0.5^a^ | 15.8 ± 1.9^a^ |
| Food intake (g) | 2.8 ± 0.2 | 3.0 ± 0.3 | 3.1 ± 0.3 | 3.0 ± 0.6 |
| Water intake (mL) | 4.6 ± 2.4 | 5.1 ± 2.9 | 4.4 ± 0.9 | 5.6 ± 3.1 |
| Urine volume (mL) | 1.1 ± 0.2 | 1.3 ± 0.3 | 1.0 ± 0.2 | 1.0 ± 0.3 |
| Urine pH | 5.7 ± 0.4 | 6.1 ± 0.6 | 5.4 ± 0.3 | 5.1 ± 0.1 |
| Feces weight (g) | 1.0 ± 0.1 | 1.3 ± 0.4 | 1.4 ± 0.2 | 1.3 ± 0.4 |
| **Day 29** |  |  |  |  |
| Body weight (g) | 17.8 ± 1.7 | 19.1 ± 0.7 | 17.3 ± 0.7 | 17.9 ± 1.3 |
| Food intake (g) | 3.0 ± 0.3 | 3.6 ± 0.7 | 3.7 ± 0.4 | 3.2 ± 0.3 |
| Water intake (mL) | 4.1 ± 0.6 | 4.0 ± 1.0 | 4.1 ± 0.6 | 4.1 ± 1.0 |
| Urine volume (mL) | 0.7 ± 0.3 | 0.9 ± 0.4 | 0.8 ± 0.2 | 1.0 ± 0.2 |
| Urine pH | 6.1 ± 0.3 | 6.3 ± 0.5 | 5.3 ± 0.3 | 5.5 ± 0.1 |
| Feces weight (g) | 1.2 ± 0.1 | 1.3 ± 0.3 | 1.7 ± 0.2^b^ | 1.3 ± 0.1^c^ |

^a^, *P*_genotype_ < 0.01; ^b^, *P* < 0.05 for control + vehicle *vs* iKsp-*Pkd1^-/-^* + vehicle by two-way ANOVA with Tukey’s *post hoc* test; ^c^, *P* < 0.05 for iKsp-*Pkd1^-/-^* + vehicle *vs* iKsp-*Pkd1^-/-^* + BB-FCF by two-way ANOVA with Tukey’s *post hoc* test. Data are shown as mean ± SD (*n*=3-7).

**Table S2** | **Primer sequences used for gene expression analysis by qPCR.**

| **Gene of interest** | **Forward primer sequence** | **Reverse primer sequence** |
| --- | --- | --- |
| *18S* | 5’-GTAACCCGTTGAACCCCATT-3’ | 5’-CCATCCAATCGGTAGTAGCG-3’ |
| *Atp2b4* | 5’-ATCTGCAGGGTTCCCAGATA-3’ | 5’-CTTAATGGACCTGCGAAAGC-3’ |
| *Calb1* | 5’-CCACAGGAAACGAGGCATTTT-3’ | 5’-ATTTCCGGTGATAGCTCCAA-3’ |
| *Cldn16* | 5’-GTTGCAGGGACCACATTAC-3’ | 5’-GAGGAGCGTTCGACGTAAAC-3’ |
| *Cldn19* | 5’-GGTTCCTTTCTCTGCTGCAC-3’ | 5’-CGGGCAACTTAACAACAGG-3’ |
| *Cnnm2* | 5’-GGAGGATACGAACGACGTG-3’ | 5’-TTGATGTTCTGCCCGTACAC-3’ |
| *Havcr1* | 5’- GGAAGTAAAGGGGGTAGTGGG-3’ | 5’- AAGCAGAAGATGGGCATTGC-3’ |
| *Slc8a1* | 5’-TCGGTGCCAGACACATTTGC-3’ | 5’-CAGCATTGCTTCCAGTGACAT-3’ |
| *Slc20a2* | 5’- CGGCGTGCTGTTCATACTAA-3’ | 5’- GCAGCATAAAACAGAGGCAGT-3’ |
| *Slc34a1* | 5’-TCAGGAAGAGGAGCAAAAGC-3’ | 5’- AAAGGAAAGCCAGCATCAGA-3’ |
| *Slc34a3* | 5’-GTGGTCAGCAGCTTTCTCAA-3’ | 5’- ACAGCACCACATTGTCCTTG-3’ |
| *Slc41a1* | 5’-TCCCTGATGGCCACTTTAGC-3’ | 5’-GATCATACCCAGGACCAAGGAG-3’ |
| *Slc41a3* | 5’-TGAAGGGAAACCTGGAAATG-3’ | 5’-GGTTGCTGCTGATGATTTTG-3’ |
| *Trpm6* | 5’-AAAGCCATGCGAGTTATCAGC-3’ | 5’-CTTCACAATGAAAACCTGCCC-3’ |
| *Trpm7* | 5’-GGTTCCTCCTGTGGTGCCTT-3’ | 5’-CCCCATGTCGTCTCTGTCGT-3’ |
| *Trpv5* | 5’-TGCTCAACTTGTTCATTGCCAT-3’ | 5’-CCACAGGAAACGAGGCATTTT-3’ |

**Figure S1 | BB-FCF treatment does not change fecal Mg^2+^ and Ca^2+^ levels in iKsp-*Pkd1^-/-^* mice.** On day 22, no significant differences were present between any of the groups for fecal Mg^2+^ excretion. *P_genotype_* = 0.3, *P_treatment_* = 0.8, *P_interaction_* = 0.9 (*n*=5-7) (A). Fecal Ca^2+^ excretion on day 22 was also not different between any of the groups. *P_genotype_* = 0.6, *P_treatment_* = 0.1, *P_interaction_* = 0.8 (*n*=5-7) (B). On day 29, no significant differences were present in fecal Mg^2+^ excretion between iKsp-*Pkd1^-/-^* mice and control mice or after BB-FCF treatment. *P_genotype_* = 0.8, *P_treatment_* = 0.8, *P_interaction_* = 0.3 (*n*=5-7) (C). Fecal Ca^2+^ excretion was also not significantly different between any of the groups on day 29. *P_genotype_* = 0.4, *P_treatment_* = 0.4, *P_interaction_* = 0.07 (*n*=5-7) (D). Data are shown as mean ± SD and analyzed by two-way ANOVA.

**Figure S2 | Serum and urinary Na^+^ and K^+^ levels after BB-FCF treatment in iKsp-*Pkd1^-/-^* mice.** Serum Na^+^ was not significantly different between any of the groups. *P_genotype_* = 0.4, *P_treatment_* = 0.5, *P_interaction_* = 0.2 (*n*=5-7) (A). Urinary Na^+^ excretion was significantly lower in iKsp-*Pkd1^-/-^* mice. *P_genotype_* = 0.04, *P_treatment_* = 0.9, *P_interaction_* = 0.8 (*n*=3-6) (B). Serum K^+^ was significantly increased by BB-FCF treatment, but not affected by genotype. *P_genotype_* = 0.2, *P_treatment_* = 0.01, *P_interaction_* = 0.05 (*n*=5-7) (C). Urinary K^+^ excretion was not significantly different between any of the groups. *P_genotype_* = 0.8, *P_treatment_* = 0.4, *P_interaction_* = 0.2 (*n*=5-7) (D). Data are shown as mean ± SD and analyzed by two-way ANOVA.
